# Beatson International Cancer Conference: Genomic Regulation and Cancer

**DOI:** 10.1038/sj.bjc.6600146

**Published:** 2002-03-04

**Authors:** K E Gordon, A P MacLaren, N I Barr

**Affiliations:** Beatson Institute for Cancer Research, CRC Beatson Laboratories, Garscube Estate, Switchback Road, Glasgow G61 1BD, UK

## Abstract

*British Journal of Cancer* (2002) **86**, 665–668. DOI: 10.1038/sj/bjc/6600146 www.bjcancer.com

© 2002 Cancer Research UK

## 

Cancer development and progression is a multi-stage process involving dynamic changes within the genome. Common features of cancer cells include uncontrolled proliferation, self-sufficiency in growth, escape from cell death and the acquisition of immortality. Understanding the different molecular routes that lead to a malignant phenotype is the key to identifying specific molecular targets to selectively kill cancer cells.

This meeting concentrated on the contribution of transcription modulators, oncogene activation and genomic instability to the cancer phenotype. This led onto the impact of microarray technology on genetic profiling of the disease and how this combined knowledge is being exploited to improve diagnosis and develop novel anti-cancer drugs.

## TRANSCRIPTION MODULATORS AND ONCOGENES

Deregulation of gene expression is a common feature in all cancer cells and is dependent on extensive chromatin remodelling within the genome. Local chromatin structure is strongly influenced by the post-translational modification of the core histones. Tony Kouzarides (Cambridge) talked about chromatin modifying enzymes, focusing specifically on the regulation of Lysine 9 (K9) on histone H3. Using both mammalian and yeast models he described how the transcriptional repressor pRb silences gene transcription in a two stage process. Initially pRb and a histone deacetylase protein (HDAC) function to deacetylate K9 on H3. pRb subsequently interacts with the Suv39 and HP-1 proteins and K9 on H3 is methylated as a secondary step. This talk gave an insight into how pRb, depending on the proteins it associates with, can function to either deacetylate or methylate chromatin.

Pier Giuseppe Pelicci (Italy) addressed the function of the retinoic acid receptor (RAR) which can function either to repress transcription or in the presence of ligand, to mediate acetylation through recruitment of co-activators. His talk focused on acute myelocytic leukaemias, which are characterised by the presence of RAR mutants. In some sub-groups of leukaemias the mutant RAR-fusion protein, generated by translocation, results in a block in differentiation and inhibition of apoptosis through association of the RAR-fusion protein with an HDAC protein. However, the vast majority of these leukaemias are sensitive to treatment with retinoic acid (RA) who's purpose is to disrupt the HDAC–RAR-fusion interaction resulting in activation of genes necessary for the differentiation process. In contrast to this, other subgroups of leukaemias where a distinct RAR-fusion protein is generated are no longer sensitive to RA alone. This is due to RA being unable to displace the HDAC protein from this specific RAR-fusion resulting in a continued block in differentiation. Pilot studies in acute myeloid leukaemia (AML) patients combining RA treatment with HDAC inhibitors were observed to induce differentiation in all the cases tested and has lead to the hypothesis that HDACs may be putative targets for differentiation treatment of AMLs that are resistant to RA treatment alone. Both of these talks gave supporting evidence that defects in chromatin modification may contribute to a number of human malignancies. Therefore, understanding the complexes regulating the state of chromatin in the cell may, as in the case of AML, modify our current treatments for certain groups of cancers.

A hallmark of cancer is the activation of specific subsets of genes involved in promoting proliferation. Consequently, the activation of a catalogue of transcription factors have been associated with cancer. The AP-1 transcription factor has been implicated in a number of biological processes and Erwin Wagner's (Vienna) talk described the function of AP-1 in mouse development and cancer. Studies using conventional and conditional knock-outs have revealed that some components of the AP-1 complex such as c-Fos, FosB and JunD are dispensable for embryonic development, whereas others like c-Jun, JunB and Fra-1 are essential for embryonic development or adult viability. c-Jun was demonstrated to be essential for the proliferation of fibroblasts and hepatoblasts, whereas JunB functions as a negative regulator of proliferation through transcriptional activation of the cyclin dependent kinase inhibitor p16. In contrast to the role for Jun proteins in cell proliferation, the Fos proteins were described by Wagner as clear determinants of bone development. Previous *in vitro* studies have demonstrated that c-Fos, unlike Fra-1 or Fra-2 has the ability to transform fibroblasts in culture and this more tumorigenic phenotype of the Fos proteins was mirrored in these transgenic mouse studies. Fra-1 transgenic mice developed osteosclerosis due to enhanced differentiation of bone matrix whereas c-*fos* transgenic mice develop osteosarcomas.

Tom Curran (Memphis) also addressed the role of *fos* in cellular transformation. This work was based on studies demonstrating a reduction in tumour progression and invasion in a TPA skin model performed in c-*fos* knock out mice. Using a transcriptional profiling approach he has focused on isolating *fos* target genes that are responsible for this transformed phenotype.

While disruption of the control of proliferation is important in tumorigenesis, the inappropriate survival of cancer cells also plays a major role in this process. The NFκβ transcription factor is an anti-apoptotic factor that plays an important function in tumour development by providing a link between inflammation and cancer. Several studies have suggested that chronic inflammation during cancer treatment may activate NFκβ providing a survival signal that can contribute to resistance to specific cancer therapies. Michael Karin (California), the keynote speaker, used NFκβ to introduce the concept of modulating transcription factor activity to enhance the cancer cell's response to apoptosis inducing drugs. John Sedivy (Rhode Island) expanded this concept by describing a novel regulation of NFκβ by the Raf kinase inhibitor protein (RKIP). Microinjection studies revealed that RKIP neutralising antibodies can activate NFκβ, an effect that is independent of the previously described role for RKIP in inhibiting Raf/MEK signalling. Sedivy demonstrated that RKIP inhibited IKKα and IKKβ kinase activities, potentially via a direct interaction with the upstream kinases TAK1 and NIK1. Studies with prostate cancer cell lines revealed that resistance to apoptosis correlated with loss of RKIP expression in these tumour models. This suggests that RKIP functions in blocking the survival signal propagated from NFκβ.

Ron Hay (St Andrews) further described a role for NFκβ activation in cancer cells. Hodgkins lymphoma (HL) is characterised by constitutive NFκβ activity which arises from at least three known mechanisms. In some cases it is due to EBV transformation. Other non-EBV transformed HL's achieve constitutive NFκβ activity due to deletion of the NFκβ inhibitor Iκβα, or constitutive activation of the IKKα and IKKβ kinases. Studies with a super-repressor of the NFκβ inhibitor Iκβα, result in the induction of apoptosis in HL derived cell lines suggesting that resistance to cell death may be due to constitutive NFκβ activity. Microarray analysis is currently being applied to HL cell lines that are sensitive to apoptosis and those that show resistance to apoptosis to identify potential activators of NFκβ. Both of these talks gave insights into how the modulators of NFκβ are being targeted in the context of the cancer cell to understand how these cells avoid apoptotic signalling events.

## GENOMIC INTEGRITY: DNA REPAIR AND TELOMERE MAINTENANCE

Loss of genomic integrity is a primary feature of cancer. Recent evidence suggests that defects in DNA maintenance pathways and telomere dysfunction promote genomic instability and drives development of the disease. Stephen Jackson (Cambridge) focused on key players involved in the detection and repair of double stranded breaks (DSB). Clues to the role of the DNA damage sensors, ATM (ataxia telangiectasia mutated) and ATR (ataxia telangiectasia related) kinases, their cell cycle targets and role in DSB repair were aided by the identification of three genes involved in cancer prone human disorders. The ATM gene in ataxia telangiectasia (AT), the MRE11 gene in an ataxia telangiectasia-like disorder (ATLD) and defects in the NSB1 gene which are associated with the Nijmegen breakage syndrome. The common features of these three conditions include hypersensitivity to ionising radiation, S-phase checkpoint defects, impaired telomere function and increased genomic instability. The NSB1 protein is one of the components of a larger complex, RAD50/MRE11/NSB1 which is involved in ‘cleaning up DSB’ in preparation for repair. Evidence obtained from deletion analysis of the analogous yeast complex (XRM) demonstrated its involvement in S-phase control in response to replication stalling and DSB. This led to a model whereby this complex, common to both homologous recombination (HR) and non-homologous end joining (NHEJ) repair pathways, has a role in amplification of weaker DNA damage signals. In response to these weak signals the XRM complex is recruited to the site of the break and the nuclease activity of Mre11 leads to recruitment and activation of Tel1p, the yeast ATM homolog, possibly by a co-factor analogous to the DNA targeting role of Ku70-Ku80 in DNA-PK complex. Tel1p then functions to phosphorylate its targets both within this complex and downstream cell cycle targets such as p53. This model helps explain the observation that cells isolated from AT and ATLD patients are hypersensitive to low doses of radiation.

Telomere maintenance is essential for tumour cells to by-pass replicative senescence and become immortal. Approximately 80% of human tumours and 99% of immortal cells stabilise their telomeres by activation of the enzyme telomerase. One of the current models suggests that during the proliferative stages of tumour development there is progressive telomere errosion that leads to short dysfunctional telomeres. In the absence of senescence and DNA damage checkpoints this telomere dysfunction drives genomic instability. The reactivation of telomerase, restores chromosomal stability by maintaining short by stable telomeres and drives the immortal phenotype. Maria Blasco (Madrid) provided *in vivo* data from mice deficient in the telomerase RNA component (Terc^−/−^) and compound knock-outs with tumour prone backgrounds to support this model. Interestingly late generation Terc^−/−^ mice with critically short telomeres were also relatively resistant to chemically induced skin papillomas, leading to the hypothesis that critically short telomeres triggers a DNA damage response and activates p53 preventing tumour progression via growth arrest or apoptosis. However, a potential pitfall of this approach is that blocking telomerase may lead to the activation of alternative telomere maintenance (ALT) pathways or may require an intact p53 response.

Murray Robinson (Amgen) presented encouraging data on the molecular response to telomerase inhibition. The introduction of an inducible telomerase dominant negative into two p53 deficient tumour cell lines with differing telomere lengths, had very distinct outcomes. In A431 cells, which have short telomere lengths, apoptosis was induced after only 3–4 days with no evidence of activation of ALT pathways. In contrast, 293T cells which have varying telomere lengths displayed reduced viability but no induction of apoptosis and reactivated telomerase. Using microarray analysis the growth arrest and DNA damage (GADD) genes, GADD45 and GADD153, were found to be up-regulated prior to the activation of apoptosis in A431 cells. GADD45 gamma was preferentially induced in A431 cells in response to telomere inhibition and it induced the expression of GADD153, which mediates the apoptotic responses or growth arrest when GADD153 is knocked out. In contrast, 293T cells constitutively express GADD45 gamma and have adapted to bypass this pathway to cell death. 293T cells harbour the adenovirus E1B 19K protein which is an anti-apoptotic homologue of Bcl-2 and therefore may not be typical of most human carcinomas. However, the clinical significance of this p53-independent GADD mediated apoptosis pathway will depend on how well utilised this pathway is in p53 deficient tumours *in vivo*. If the majority of cancers show a similar response to A431 cells then telomerase inhibitors have great potential, but if most tumours display properties similar to 293T cells then a combinational therapeutic approach may be necessary.

Direct links between telomere dysfunction and the DNA repair pathways are starting to emerge. In yeast and mammalian systems components of the DNA repair pathways, RAD50/MRE11/NSB1, DNA-PK and its targeting component Ku are associated with the ends of chromosomes and relocalise to the sites of double strand breaks. Defects in the DSB repair pathway can lead to dysfunctional telomeres as alluded to by Stephen Jackson. To address the role of these DNA repair proteins in the context of telomere maintenance, Maria Blasco examined telomere lengths in mice deficient in Ku86 or DNA-PKcs. Ku86 deficient cells display a more acute telomeric phenotype than DNA-PKcs deficient cells showing higher rates of telomere-telomere fusions. Surprisingly, Ku86 deficient cells have slightly elongated telomere ends at the fusion points compared to DNA-PKcs. It was proposed that mammalian Ku86 acts to suppress telomere elongation via direct interactions with the telomere binding proteins TRF1 and TRF2 at T-loop structures creating a tight cap around the telomere end. In the absence of Ku86 the cap is slackened and allows telomerase and DNA repair activities access to elongate and fuse telomeres respectively. This suggests that dysfunctional telomeres can be generated in different ways. Telomeric attrition results in short telomeres at the fusion points whereas loss of telomere protective function via loss of Ku86, DNA-PKcs results in long telomeres at the fusion points.

A theme which persisted throughout the talks on DNA damage and telomere function is the need to rethink our approaches to therapeutic strategies. While previously the main thrust of research has focused on modulating p53 activity in tumours, data is now beginning to emerge suggesting that p53, DNA damage pathways and telomere maintenance are all interlinked processes and that we should be considering combined intervention approaches to target the cancer cell.

## THERAPEUTIC TARGETS

The selective activation of telomerase and inactivation of p53 in the majority of cancers has lead to therapeutic strategies based on inhibition of telomerase (as discussed above) in addition to reactivating p53. The p53 protein plays a central role in maintaining genomic integrity by responding to a variety of DNA damage signals and inducing cell cycle arrest or apoptotic cell death. Testimony to the success of p53 as a tumour suppressor is that the vast majority of human tumours have either a mutated p53 gene or defects within p53 signalling pathways. Preliminary therapies were based on reactivating p53 directly, but more recently the focus has been on the isolation of small molecules which mimic the function of proteins that stabilise p53. Karen Vousden (Frederick) described this approach for the identification of modulators of p14^Arf^ and David Lane (Dundee) for Mdm2 E3 ligase activity modulators.

One exciting aspect of these therapeutic based talks was the development of combined strategies to target more than one key pathway in the cell. If anything our previous experience in this field has demonstrated that the complexity of changes present within the cancer cell require a more intellectual approach to this problem if we are to overcome it.

## TRANSCRIPTIONAL PROFILING

Global analysis of gene expression through microarray analysis was a common theme throughout the entire meeting. Conventionally, analysis of differences in expression of genes has been performed by Northern blot analysis or quantitative PCR of reverse transcribed RNA. However, with the advent of DNA microarray technology it is now possible to compare patterns of global gene expression simultaneously rather than focusing on individual genes, thus facilitating a more inclusive experimental approach to studying gene transcription on a genome wide scale. In cancer, this technology is very powerful because of the multi-step nature of tumorigenesis where a number of genes must be disrupted for malignancy. Therefore, using microarray hybridisation analysis this complex pattern of gene expression can be monitored simultaneously. This will allow the classification of tumours according to their gene expression profiles and has the potential for the diagnosis of cancer and prediction of responses to therapy.

Microarray analysis has now become common practice in many research laboratories, however one of the major challenges with this technology is still the interpretation of such complex and vast data sets. David Botstein (Stanford, CA, USA) and others have used clustering techniques to compare patterns of gene expression to each other and to combine patterns that are the most similar (hierarchical clustering). For example, clustering genes that have similar expression patterns during cell cycle progression into a phase ordered map reveals clusters of periodicity allowing the assignment of different clusters of genes into different stages of the cell cycle (a so-called ‘phasogram’). This has the added advantage of allowing the function of previously unknown genes to be inferred based on clustering with other genes.

In cancer tissue samples, microarray analysis and clustering algorithms have also been used to classify tumours in a biologically significant way based on their expression profiles, often correlating with clinical outcomes (‘class prediction’). Todd Golub (Boston, MA, USA) has extended this approach using DNA microarrays to subclassify human acute leukaemias. Acute lymphoblastic leukaemia (ALL) and acute myeloid leukaemia (AML) are molecularly distinct but morphologically similar resulting in diagnostic problems. The correct diagnosis of ALL and AML is critical for successful treatment and to minimise toxicity since different chemotherapy regimens are used to treat each class. Using DNA microarrays, these tumours were distinguished from each other at the gene expression level. Furthermore, ALL subgroups could be further classified according to chromosomal rearrangement status at the MLL gene, which comprise a distinct type of acute leukaemias which share characteristics of both ALL and AML. ALLs harbouring MLL rearrangements were shown to differ by greater than 1000 genes. By applying clustering algorithms to the data, three groups of genes which were differentially expressed were found, distinguishing MLL from ALL and AML.

This ability to sub-classify leukaemia, and other cancers such as breast and lung, according to their gene expression profile has enormous clinical potential in the diagnosis of cancer and also in the prediction of the response to chemotherapy. Indeed, microarrays are currently being tested for their use in a clinical setting to predict the optimal chemotherapeutic programme for individual patients. Edwin Clark (Cambridge, MA, USA) described the use of pharmacogenomics to study the global gene expression in relation to therapeutic response. As a proof of concept, 51 ovarian cancer tissues were profiled retrospectively for the expression of 30 000 human genes by microarray analysis to identify expression markers which correlate with clinical outcome in response to a combination treatment of taxol and platinum. Using a series of clustering algorithms, Clark was able to identify genomic markers which predicted response to therapy with an accuracy of 85–95%, thus allowing alternative therapy and care for those patients who were predicted to be ‘non-responders’ to taxol and platinum treatment.

It is now becoming more realistic to imagine that the implementation of DNA microarray based diagnoses to a single patient could become routine. However, several problems are associated with this type of analysis in the clinical setting, such as high cost, the complexity of the techniques involved as well as the inherent instability of the RNA molecule. Joe Gray (San Francisco, CA, USA) described a relatively new technique which may overcome at least one of these problems; namely the reliance on good quality RNA. He described using DNA to perform quantitative measurements of DNA copy number in breast cancer by array comparative genomic hybridisation (CGH). Genomic instability is a hallmark of cancer and changes in copy number of some genes occur as tumours more to more invasive phenotypes. The length of the telomere plays an important role in development of genomic instability, the transition of tumours through telomeric crisis results in structural changes in the chromosome and genomic instability. In view of the random nature of chromosomal instability resulting from translocations, mitotic breaks and end-to-end fusions of chromosomes during telomeric crisis, most individual cancers are highly variable in terms of the numbers and types of genome abnormalities involved. This poses a big challenge for conventional genome copy number analysis using CGH and fluorescent *in-situ* hybridisation (FISH) because this technique is too labour intensive to apply across the whole genome. Array CGH by comparison has the potential to cover much of the genome in one experiment thus achieving increased mapping resolution of gene amplifications and deletions. Gray's recent data using a 500 element BAC array has highlighted the variability of abnormalities between primary breast tumours which are clinically similar, however a number of genes do crop up frequently, and these may represent attractive targets for therapeutic intervention. For example, *CMYC*, *ERBB2* and *CCND1* are amplified in more than 20% of advanced breast cancers and they are all currently being investigated for targeted therapeutics to specifically knock out their functions. However, the spectrum of genomic abnormalities found using the CGH array system varied substantially among tumours extending the idea for personalised therapy to be used in the clinic. Gray also showed that there was good correlation between genome copy number and gene expression across a panel of 60 cell lines establishing the relevance of this technique to gene expression within the cell.

It is clear that the introduction of microarray-based technology into the field of cancer research has greatly accelerated our understanding of the processes involved in the development of tumours by allowing the simultaneous monitoring of the gene expression of thousands of genes. This will inevitably result in better diagnosis and novel therapies to target aberrantly expressed genes in complex biological processes.

Whilst we are still a long way from appreciating all of the complex changes in gene expression that contribute to the cancer cell phenotype, the enthusiasm generated during this meeting provided enlightening prospects for unique strategies to fully comprehend and more importantly combat cancer.

